# *Candida auris* cluster in a large third level Italian hospital: a case series

**DOI:** 10.1016/j.ijregi.2024.100468

**Published:** 2024-10-09

**Authors:** Antonio Di Lorenzo, Francesco Triggiano, Marco Lopuzzo, Luigi Piccolomo, Marco Triggiani, Salvatore Grasso, Pasquale Stefanizzi, Silvio Tafuri, Lidia Dalfino, Giuseppina Caggiano

**Affiliations:** 1 Interdisciplinary Department of Medicine, Hygiene Unit, University of Bari Aldo Moro, Bari, Italy; 2 Department of Emergency and Organ Transplantation, Anesthesia and Intensive Care Unit, University of Bari Aldo Moro, Bari, Italy

**Keywords:** Intensive care unit, Sentinel pathogen, Outbreak

## Abstract

**Objectives:**

*Candida auris* has been circulating since 2019 in Northern and Middle-Italy regions.

**Design:**

This report details the first three cases of *C. auris* isolation in Puglia (Southern Italy), which occurred as a nosocomial outbreak.

**Results:**

The first *C. auris* specimen was isolated on April 19, 2024 from a blood culture obtained from a 55-year-old male (GM) in an intensive care unit (ICU) of Bari Policlinico General Hospital. The patient had been admitted on April 06, 2024 due to subarachnoid hemorrhage. On April 12, 2024, a blood sample tested positive for multidrug-resistant *Klebsiella pneumoniae*, requiring isolation and wide-spectrum antibiotic therapy. Exitus occurred on April 17, 2024, but 2 days later, a pre-mortem blood sample tested positive for *C. auris*, leading to epidemiologic investigation in the whole ICU.

A second case was therefore identified (SD), a 36-year-old male admitted on March 21, 2024, shortly after undergoing a dental procedure in Tirana (Albania). Due to the timing of admission, he was identified as the index case.

A third case was later found on April 29, 2024 (CM), a 52-year-old woman with a history of acute myeloid leukemia located in the same ICU but on a different floor.

A link of transmission was identified via environmental sampling, as the medics’ common telephone tested positive for *C. auris* contamination.

**Conclusion:**

The pathogen's circulation has shown to be inapparent and difficult to track, making it a potential threat even for facilities outside of known high-risk geographical areas. ICUs should be monitored routinely.

## Highlights

• This is the first nosocomial *Candida auris* outbreak to be identified in Southern Italy.
• The outbreak occurred in an intensive care unit.
• The index case had been recently hospitalized in Albania.
• Health care workers may have had a role in the transmission of *C. auris*.
• Infection prevention and surveillance should be strengthened.

**Strengths and limitations of the study**

*Strengths:*

• This is the first outbreak of *C. auris* to be reported in a Southern Italy health care facility.
• Outbreak description based on laboratory data and epidemiologic investigation.

*Limitations:*

• When the outcome occurred, the hospital was not enforcing routine C. auris surveillance.
• The index case was identified retrospectively.

Alt-text: Unlabelled box

## Introduction

*Candida auris* is an emerging opportunistic yeast first isolated in Japan in 2009 [1].

It belongs to the genus *Candida*, a commensal organism commonly found in the skin and gut microbiota but capable of causing invasive disease. Invasive candidiasis represents a significant health care-associated fungal infection, with a case fatality rate exceeding 70%. Although the prevalence of these organisms varies considerably depending on geographical location, the most common *Candida* species include *C. albicans, C. glabrata*, and *C. parapsilosis*, the next last responsible for nosocomial outbreak [2]. During the last decade, *C. auris* has emerged globally as a health care-associated fungal pathogen characterized by innate resistance to different antifungal classes [3]. It is typically responsible for infections in high-risk patients, intensive care unit (ICU) patients being the most frequently involved; its characteristics of multidrug resistance, mixed with these patient category's high vulnerability, account for a significant mortality of *C. auris* infections. Patients who are involved in the disease process are typically colonized in multiple cutaneous districts, including the axillary, perineal, and auricular regions [3,4]. These patients may then progress to more severe forms of invasive fungal infection.

Following its initial isolation, *C. auris* was identified in several other countries, always sharing its aggression pattern against ICU patients [[5], [6], [7], [8], [9], [10], [11], [12], [13], [14], [15], [16], [17], [18], [19], [20], [21]]. In these instances, *C. auris* caused various infections, both local and systemic. However, bloodstream infections were the most frequently observed *C. auris* invasive infections, with a 30-60% hospital case fatality rate [7,12,22].

The main risk factors for *C. auris* infection are long-term ICU stay, immune depression, general decay of clinical conditions, and prolonged wide-spectrum antimicrobial therapy [23,24]. The fungus has also got relatively high environmental resistance, being capable of surviving for long periods of time on the hands of medical staff and on inert surfaces, including medical devices employed for diagnostic and therapeutic procedures [25]. Infected patients may also spread the pathogen by breathing and coughing; although the mode of transmission has typically been reported to occur through direct contact, there is a paucity of evidence regarding long-range airborne dispersion during high-turbulence activities [26].

European *C. auris* outbreaks were studied by the European Centre for Disease Control and Prevention in a 2022 rapid risk assessment [27], which highlighted a significant rise in the overall risk starting from 2020, likely linked to the COVID-19 pandemic. It can be reasonably postulated that the respiratory symptoms exhibited by patients affected by COVID-19 may have contributed to the increased circulation of *C. auris*. Furthermore, the crowding of ICU wards with highly vulnerable patients during the pandemic increased the risk for cross-contamination, and the simultaneous presence of patients infected with different pathogens necessitated the administration of antimicrobial therapies with a broad spectrum of activity, in addition to the prolonged use of corticosteroids, which is known to be a risk factor for fungal complications [28].

In Italy, *C. auris* was first isolated in Northern Italy in 2019; in 2022, *C. auris* outbreaks had been identified in at least eight different hospitals in the same Italian region, and over 277 cases had been diagnosed. More cases were later observed in Lazio and Emilia-Romagna (Middle Italy), and therefore, nationwide surveillance was recommended by the European Centre for Disease Control and Prevention [27]. The Italian Ministry of Health also defined the guidelines for health care facilities to follow in case of *C. auris* isolation [29].

This report describes a case series that occurred in April 2024 at Bari Policlinico General Hospital in Puglia (Southern Italy).

## Location characteristics

Bari Policlinico General Hospital is the largest hospital in Southern Italy, hosting 1,550 beds. It has a pavilion-based structure, where wards are located in separate buildings. The two ICU facilities are especially isolated from other wards, having dedicated pavilions.

Since 2020, Bari Policlinico has activated a special program named control room. It represents an organizational overseeing unit focused on infection prevention and control. All wards may ask the control room staff for consultation regarding infection control procedures and notify them of infectious disease cases. In addition, the control room manages health care-associated infection surveillance and epidemiologic investigations within the hospital.

The case series was located in the “Anestesia e Rianimazione II Universitaria” ward (University Anesthesia and Reanimation II, henceforth RIA), a university ICU. The facility is located in a three-story pavilion, with one underground floor functioning as medical storage and clinical records archive. The ground floor hosts the building's reception and security posts. The two upper floors are dedicated to 16 bed stays (eight per story). Each of them has a filter zone with locked doors separating the actual ward from common spaces, as well as locker rooms for the staff to change in.

Beds are located in a single intensive care room per floor, with physical barriers separating them to prevent pathogen transmission among patients. Each intensive care room also hosts a station dedicated to medical staff, with a corded telephone, computers, and a cabinet containing clinical records of currently hospitalized patients. This kind of arrangement aims to make it easy for medical personnel to rapidly intervene in case of emergency while also continuing to monitor patients and providing at least some degree of isolation to them. Each floor also has confinement boxes with transparent plexiglass walls dedicated to isolating infected patients.

Policlinico's Hygiene and Public Health Unit hosts the Environmental and Food Hygiene laboratory equipped for the isolation of *C. auris* [2]. It is responsible for conducting microbiologic investigations on a range of environmental matrices, including food, water, air, and surfaces. Following episodes of health care-associated infection, the laboratory collaborates with the control room unit to prevent health care-related infections through environmental investigations. This is done to identify the possible source of infection and the environmental reservoir.

## Laboratory methods

In the period between April 19, 2024 (the first case) and May 01, 2024, 176 surfaces in the RIA unit were sampled. Specifically, the high-touch surfaces of each box were assessed (touch screen multiparametric monitors, touch screen monitors for assisted ventilation, bed rails, infusion pumps, bed keypad, and medication preparation rack), as well as the common surfaces of the department (hand disinfectants dispensers, computer keyboards and mice, ultrasound probes, medicine cabinet handles, and telephone).

A total of 201 samples were collected, comprising 25 samples from health care personnel hands and 176 samples from high-touch surfaces. The surfaces were sampled using sterile swabs soaked in distilled water. A single swab was used for each health care worker, applied to both hands. All swabs were transported to the laboratory in refrigerated containers at a temperature of +4°C and were tested immediately upon arrival.

To detect the presence of *C. auris* nucleic acid, a molecular method was performed. The research was carried out directly on clinical and environmental swabs without any extraction using the kit AurisID®, Olm Diagnostics. The protocol is based on 40 amplification cycles, in which it is possible to detect <1 copy per reaction. The test should be interpreted as positive if amplification occurs within 40 cycles.

Swabs were mixed with 0.5 ml distilled water and shaken for 30 seconds to facilitate cell detachment. For molecular analysis, a master mix was prepared according to the manufacturer's instructions: 10 µl AurisID qPCR Master Mix, 2 µl Auris Prime Probe Mix, and 1.8 µl RNase/DNAse-free water. Since DNA extraction was not performed, the internal extraction control (0.2µl/sample) was included in the mix. Subsequently, 6 µl of each sample was added to 14 µl of the master mix, and the polymerase chain reaction (PCR) reactions were performed on a CFX96 Touch Deep Well Real-Time PCR System (Bio-Rad, Hercules, California, USA). Positive and negative controls supplied with the kit were included.

Clinical surveillance was performed on all hospitalized patients by swabbing their inguinal/axillary cavity once a week, with swabs also taken from other body sites, such as wounds, nasal swabs, pharyngeal swabs, and tracheal bronchial aspirates, where deemed appropriate.

## Outbreak description

### Case one: GM

On April 19, 2024, the RIA unit staff notified the control room regarding a *C. auris* isolation in one of their patients. The patient was GM, a 55-year-old white Caucasian male who had accessed the hospital's emergency room on April 05, 2024 due to a headache occurring during a hypertensive crisis; the patient was treated for hypertension and reported smoking habit. Due to the worsening of symptoms, GM underwent cranium computerized tomography (CT) and angio-CT, which identified a subarachnoid hemorrhage associated with an aneurysm of the right mean cerebral artery.

On April 06, 2024, GM was transferred to the Neurosurgery Unit for surgical clipping of the bleeding artery. After surgery, the patient was hospitalized in the RIA unit for monitoring of clinical conditions; he was located in bed 7, on floor one. On April 12, 2024, GM had a fever peak with sensory alteration and desaturation, and the arterial blood gas test highlighted the worsening of gas exchanges.

A blood culture was performed, resulting in a positive for multidrug-resistant (MDR) *Klebsiella pneumoniae*. A consultation was requested to the Infectious Disease Unit's staff, who recommended antibiotic therapy with ceftazidime-avibactam and aztreonam. This therapy was immediately started. The patient was transferred to the isolation box (bed 4) on floor one, and contact isolation procedures were enforced. Despite the therapy, clinical conditions of GM kept worsening until exitus occurred on April 17, 2024.

On April 19, a blood sample taken shortly before death tested positive for *C. auris*, leading to the control room being notified. On the same day, the control room staff transmitted the notification to regional health authorities, as per Italian regulation [30], and initiated an epidemiologic investigation to identify possible new cases of *C. auris* infection. The investigation was curated by the Control Room's and Laboratory of Environmental and Food Hygiene staff.

First, the story of GM was studied. No clues of travels in geographical areas known for circulation of *C. auris* were found, and the patient had not been hospitalized in the previous 6 months. The patient had not been to Italian regions with previous history of *C. auris* outbreaks, either.

Subsequently, multiple skin swabs were collected from each patient currently residing on the first floor of the RIA unit's pavilion. In addition, high-touch surfaces on the same floor, including technological equipment, cabinets, and medical records, were swabbed and tested for contamination with *C. auris* (26 by surface and 48 by patient). Following this investigation, only one patient tested positive for *C. auris.* Further details are provided in the subsequent section. The first-floor landline tested positive for contamination, as did various surfaces in box 4 (bed rails and medication preparation rack) and box 8 (touch screen multiparametric monitors, touch screen monitors for assisted ventilation, bed rails, infusion pumps, and bed keypad). The environment was disinfected rapidly using hydrogen peroxide *fast* at a concentration of 1.5% while the patient underwent skin washing with chlorhexidine.

### Case “zero”: SD

On April 19, 2024, immediately after the notification of *C. auris* positivity, the same yeast was identified in skin swabs taken from the axillary and perineal regions of SD, a 36-year-old Caucasian white male. SD had no known history of disease and had accessed Policlinico's emergency room on March 21, 2024. After investigation, it was discovered that SD had recently been to Tirana (Albania) to undergo a dental care procedure, which occurred on March 13, 2024.

On the very same day, SD suffered a ventricular fibrillation episode with cardiac arrest and was rescued by the Albanian emergency service. He was treated with cardiopulmonary resuscitation and adrenalin, transferred to Tirana General Hospital and hospitalized in ICU, suffering a new ventricular fibrillation on March 14, 2024. After defibrillation and coronarography, SD was diagnosed with an occlusion of the anterior descending coronary artery, and double antiplatelet therapy was initiated.

On March 16, 2024, Tirana General Hospital's staff contacted Bari Policlinico's RIA unit to arrange the patient's transfer to his hometown. He was finally transported via helicopter on March 21, 2024 and hospitalized with the diagnosis of “post-anoxia coma in a patient who recently underwent dental care procedures.” Upon arrival, the patient was sedated, curarized, ventilated via flowmetry-controlled ventilation, and catheterized; central venous access was obtained, and blood samples were taken for chemicals and microbiologic testing. Vital parameters were normal, but a fever peak was observed, and the patient was therefore located in an isolation box (bed 8), contact isolation being enforced.

The blood culture was positive for both carbapenem-resistant *K. pneumoniae* and MDR *Acinetobacter baumannii*. After specialist consultation, antibiotic therapy with cefiderocol, ampicillin-sulbactam, and fosfomycin was started. New fever episodes were observed in the next few days. Fosfomycin therapy was suspended on March 29, 2024.

On March 30, 2024, blood cultures tested positive for *C. parapsilosis*. Consultation was once again requested, and caspofungin and daptomycin were added to the ongoing therapy. On April 05, 2024, the fever regressed, and all antimicrobial therapies were suspended. On April 10, 2024, a new blood culture tested positive for MDR *K. pneumoniae*, and ceftazidime-avibactam therapy was initiated.

On April 16, 2024, a molecular rectal swab identified kpc and ndm carbapenem-resistance genes, and aztreonam was added to therapy. On April 18, 2024, rectal swab also highlighted *C. albicans* colonization. On April 19, 2024, SD was re-evaluated by the Infectious Disease Unit's staff, and the current antibiotic therapy was suspended and replaced with imipenem-relebactam therapy. Later that day, after screening, SD tested positive for *C. auris*. The case was notified to competent regional authorities.

Due to the recent travel history with hospitalization in a foreign country, SD was identified as the index case for the ongoing *C. auris* outbreak. Contact tracing was performed to identify all patients who had been hospitalized on the first floor of the RIA unit since March 21, 2024, and all staff who came into contact with either SD or GM. None of the identified patients tested positive for *C. auris*, nor did the staff.

On April 24, 2024, rooms of the General Surgery and Neurosurgery Units were swabbed since they had hosted patients who had been identified as contacts of the two cases. All spaces, however, tested negative. Moreover, 25 new environmental swabs were taken from the RIA unit's first floor. In this case, the drugs cabinet's handle and the patients’ hygiene cart tested positive for *C. auris*. The environment was disinfected using hydrogen peroxide *fast* at a concentration of 1.5%.

Due to the evidence of a nosocomial cluster, new hospitalizations were halted, and patients who were already stationed in the RIA unit were inhibited from being transferred to other wards. The two floors’ nursing staff were separated to limit the risk of between-floor transmission, and weekly screening of patients who had been treated by the same staff was planned.

Contacts of the case were immediately screened, including both patients and health care staff. Overall, nine patients and 15 staff members were screened, testing negative.

### Case two: CM

On April 29, 2024, a screening skin swab was taken from patient CM, a 52-year-old white Caucasian female hosted on the second floor of the RIA unit. The patient tested positive for *C. auris*. The screening was performed because CM was scheduled to be transferred to the Hematology and Transplant Unit.

CM has a previous diagnosis of acute myeloid leukemia treated via blood stem cell transplantation, which was followed by a relapse; CM, therefore, required routine hematologic re-evaluations. On April 16, 2024, CM underwent a blood transfusion during one of these routine assessments, and on April 17, 2024, CM accessed Policlinico's emergency room due to persistent fever with syncopal episodes and sphincteric release. Low blood pressure was detected, and noradrenalin was administered.

Blood chemical testing highlighted high inflammatory indexes, leading to the initiation of empiric antibiotic therapy with meropenem. On the same day, the patient was hospitalized in the Internal Medicine ward due to lack of space in the Hematology unit. The diagnosis at arrival was “severe sepsis in a patient with acute myeloid leukemia,” and CM was evaluated with a recommendation to undergo leucocyte and platelet concentrate transfusions, daily blood count and kidney functionality monitoring, and hemocultures during fever peaks.

In addition, on April 17, 2024, following a progressive worsening of the hemodynamic parameters, CM was transferred to the RIA unit. She was located on the second floor of the facility with a septic shock diagnosis. The patient was awake, collaborating, and apyretic upon arrival, with oxygen therapy via nasal cannula and adrenalin hemodynamic support.

On April 18, 2024, CM was tested for *C. auris* colonization/infection, with negative results. Empiric antimicrobial therapy with meropenem, vancomycin, acyclovir, and amphotericin B was started to counter the septic status and prevent viral and fungal infections. Later that day, the patient's respiratory performance plummeted, leading to an increase in oxygen therapy with a change to orotracheal intubation.

During the following days, a progressive stabilization of the hemodynamic and respiratory performance was observed, and noradrenalin support was suspended on April 26, 2024. On April 29, 2024, CM was clinically stable, on nasal cannula oxygen therapy, with normal body temperature and blood pressure. Transfer to the Hematology unit was therefore planned. However, as already stated, the recent *C. auris* cases led to a preventive fungal colonization screening. Subsequently, an axillary and inguinal skin swab was collected and subjected to microbiologic investigations by reverse transcription-PCR. CM was identified as a third case, albeit asymptomatic. The case was notified to regional authorities.

Contact tracing was performed, and both patients and staff who came into contact with CM were tested for *C. auris* colonization. Seven patients and 16 staff members were identified as possible contacts. None of them, however, tested positive. Environmental screening of the second floor of the RIA unit was also performed, with the identification of *C. auris* on the surfaces of the box 8 bed (empty, previously occupied by a patient who had been transferred to a different hospital with no symptoms of infection). On April 30, 2024, CM was transferred to the Infectious Disease Unit for isolation.

All transfers to the RIA unit's pavilion were suspended from April 19, 2024 to May 01, 2024, when no *C. auris* circulation could be detected during microbiologic surveys. SD was still hospitalized in bed 8 on the building's first floor, but all surfaces had been sanified, and resuming routine activities was deemed non-dangerous for patients’ safety. Still, isolation procedures were strengthened on SD's box. Weekly *C. auris* screening was also activated for all hospitalized patients, and screening upon arrival was decided for all patients.

## Discussion

Since no evidence of patient-to-patient transmission was found, it is plausible that *C. auris* was transmitted via the hands of health care workers (HCWs) operating in the RIA unit. This theory is corroborated by the findings of environmental microbiologic surveys: high-touch surfaces were the only ones that tested positive, and all patients were unable to move due to their clinical conditions.

The role of HCWs in hospital transmission of *C. auris* has been hypothesized by other authors due to the identification of the fungus from nasal swabs of hospital staff during an outbreak in the United Kingdom [18,31]. In this case, although HCWs tested negative for *C. auris* colonization, the pathogen's presence on high-touch surfaces necessarily implies that it was transported there by the staff.

Although hand hygiene was probably the main critical factor in determining this case series, the first case's origin remains unknown. No episodes of *C. auris* isolation were ever reported in Albania [27], but Puglia had never reported outbreaks, either. Moreover, none of the three patients came from Italian regions with previous *C. auris* isolation. Finally, the only case of candidemia was observed in a patient with high clinical risk due to a combination of recent neurosurgery, prolonged antibiotic therapy, and ongoing bloodstream infections sustained by MDR microorganisms.

This case series highlights the need to strengthen infection control procedures in European hospitals. The pathogen's circulation has shown to be inapparent and difficult to track, making it a potential threat even for facilities outside of known high-risk geographical areas. ICUs should be monitored routinely via environmental and patient testing, with special attention for patients undergoing long-term, wide-spectrum antimicrobial therapy.

In addition, the presence of *C. auris* in Policlinico General Hospital has significant implications for public health. In the future, patients transferred from this hospital's ICUs to other health care facilities will require special attention to prevent *C. auris* spread. Second, a coordinated effort from the hospital's management and regional policymakers is needed to counteract the pathogen's presence within the hospital itself by instituting specific regulations and protocols. The use of *ad hoc* measures might be particularly efficient, as well as an appropriate risk communication to health care personnel.

Finally, this case series showed how the transportation of a patient between hospitals is a critical moment for infection control. Special precautions need to be taken, including close surveillance of these patients upon arrival, especially in case of international movement from areas known for *C. auris* circulation.

## Declarations of competing interest

The authors have no competing interests to declare.
